# Conserved defense responses between maize and sorghum to *Exserohilum turcicum*

**DOI:** 10.1186/s12870-020-2275-z

**Published:** 2020-02-10

**Authors:** Xiaoyue Zhang, Samuel B. Fernandes, Christopher Kaiser, Pragya Adhikari, Patrick J. Brown, Santiago X. Mideros, Tiffany M. Jamann

**Affiliations:** 1 Department of Crop Sciences, University of Illinois at Urbana-Champaign, Urbana, IL 61801 USA; 2 Department of Plant Sciences, University of California, Davis, CA 95616 USA

**Keywords:** Sorghum leaf blight, Genome-wide association mapping, Quantitative disease resistance, Northern corn leaf blight, Exserohilum turcicum, Setosphaeria turcica, Sorghum

## Abstract

**Background:**

*Exserohilum turcicum* is an important pathogen of both sorghum and maize, causing sorghum leaf blight and northern corn leaf blight. Because the same pathogen can infect and cause major losses for two of the most important grain crops, it is an ideal pathosystem to study plant-pathogen evolution and investigate shared resistance mechanisms between the two plant species*.* To identify sorghum genes involved in the *E. turcicum* response, we conducted a genome-wide association study (GWAS).

**Results:**

Using the sorghum conversion panel evaluated across three environments, we identified a total of 216 significant markers. Based on physical linkage with the significant markers, we detected a total of 113 unique candidate genes, some with known roles in plant defense. Also, we compared maize genes known to play a role in resistance to *E. turcicum* with the association mapping results and found evidence of genes conferring resistance in both crops, providing evidence of shared resistance between maize and sorghum*.*

**Conclusions:**

Using a genetics approach, we identified shared genetic regions conferring resistance to *E. turcicum* in both maize and sorghum. We identified several promising candidate genes for resistance to leaf blight in sorghum, including genes related to R-gene mediated resistance. We present significant advancements in the understanding of host resistance to *E. turcicum*, which is crucial to reduce losses due to this important pathogen.

## Background

Translation of host plant resistance from one species to another facilitates the development of resistant varieties. Furthermore, knowledge of pathogen evolution can be informative for disease management, including host resistance. One process by which microbes become pathogens of plants is that pathogens jump from one host to a new species [1]. When a pathogen moves to a new host, knowledge from the original pathosystem can be translated to the novel crop species. Sorghum is the world’s fifth most important cereal crop, and biotic stress limits sorghum production. Host resistance is vital for the management of biotic stresses. Sorghum is closely related to maize, but less is known about biotic stress resistance in sorghum.

The foliar fungal pathogen *Exserohilum turcicum* (Pass.) K. J. Leonard & Suggs (syn. *Setosphaeria turcica* (Luttr.) K. J. Leonard & Suggs), is a pathogen of both maize and sorghum, causing northern corn leaf blight (NCLB) and sorghum leaf blight (SLB). Maize and sorghum are two of the most important cereal crops and are both susceptible to *E. turcicum*. In maize, NCLB is considered one of the most important diseases in the United States [2]. It was estimated that NCLB caused the loss of 27.9 million metric tons of maize between 2012 and 2015, the most extensive loss due to a disease. In sorghum, SLB is considered an important fungal disease. If infection occurs before emergence of the panicle, it may lead to grain yield losses of up to 50% [3]. The disease is most devastating in areas with high humidity and moderate temperatures [4]. Of concern, highly susceptible varieties have been adopted for production in some regions [5]. On maize, the disease first appears as small, tan flecks on leaves, and on sorghum, as small reddish flecks. Flecks enlarge and coalesce into long, elliptical lesions with reddish or brown borders. Borders can vary in color in both hosts depending on the genotype.

*E. turcicum* co-evolved with maize in Mexico, and subsequently jumped to cause disease on sorghum [6]. A single locus underlies host specificity on maize and a second single locus underlies host specificity on sorghum [7]. The pathogen is capable of sexual reproduction in the field, and uses a mixed reproductive strategy [8, 9]. The simple genetic architecture of host specificity and the incidence of sexual reproduction in the field makes host jumps highly likely. While genetic differentiation was observed between maize and sorghum *E. turcicum* isolates, gene flow has been observed between isolates from the two hosts, indicating that maize- and sorghum-specific isolates mate in nature [9].

The high evolutionary potential for this pathogen, characterized by the ability to undergo sexual reproduction in the field and large population sizes, emphasizes the importance of developing durable resistance, in particular, resistance that is effective in both maize and sorghum*.* While chemical control and cultural methods exist to control leaf blight, planting resistant cultivars is the most economically and environmentally friendly method of disease control [10]. Host resistance in maize has been well studied [11–16], but the relationship between resistance in the two crops is not well understood.

Both qualitative and quantitative resistance have been described in maize. Several major genes effective against NCLB have been identified, including *Ht1, Ht2, Ht3, HtN* and *ht4* [17]. Quantitative resistance has also been well-studied for NCLB with several genes being implicated including *pan1*, *ZmREM6.3*, and a caffeoyl-CoA O-methyltransferase [11–13]. However, resistance to *E. turcicum* in sorghum is not well understood. Few studies have been conducted in sorghum on host resistance to *E. turcicum* [18, 19]. Previous work has hypothesized shared resistance mechanisms between maize and sorghum, namely a highly conserved CC-NB-LRR encoding gene cluster on sorghum chromosome 5 that conferred resistance to *E. turcicum* [20]. However, no previous studies have explored genetic variation conferring resistance in both maize and sorghum.

The sorghum conversion panel (SCP) is a collection of lines where exotic lines were backcrossed for several generations to an elite line [21]. This panel includes approximately 800 converted lines that have been backcrossed with Tx406 so that the genome is largely the exotic parent with introgressions conferring early maturity and dwarfing [22]. The SCP consists of individuals from all five sorghum subpopulations. The SCP is well-suited for mapping disease resistance, as the lines are photoperiod insensitive and dwarfed, creating homogeneity to standardize disease resistance evaluations, yet sufficient allelic diversity to identify novel alleles for resistance.

Our central hypothesis is that maize and sorghum share resistance mechanisms. The objectives of this study were to i) identify loci associated with host resistance to SLB in sorghum; ii) identify candidate genes for SLB resistance; iii) compare the genetic architecture of maize and sorghum; iv) identify shared resistance mechanisms between maize and sorghum*.*

## Results

### Evaluation of the resistance to *E. turcicum*

We evaluated the SCP for SLB in 2016 and 2017 in the field and in 2018 in the greenhouse. Line was highly significant for all field environments (*P <  0.0001*) and greenhouse incubation period (*P* = 0.0464), but not for the greenhouse DLA measurements (*P* = 0.2187) (Table 1). Thus, we did not include greenhouse DLA data in any further analyses. In all field environments, we observed positive skewness, and some lines were completely resistant. For the 2018 IP data, we observed a bimodal distribution. Despite the differences in distribution between the field and greenhouse data, we found significant correlations (*P* <  0.05) between the field and greenhouse IP data, as well as a significant positive correlation (coefficient = 0.52; *P* <  0.01) between the two field-collected datasets (Table 2). Because SLB progresses after flowering, we divided the population into five sets based on flowering time to control for the relationship between maturity and disease severity. We did not find a significant correlation between flowering time and disease severity in the field environments, but there was a weak positive correlation (coefficient = 0.07; *P* <  0.10) between incubation period and flowering time.

**Table 1:** Significance of factors in mixed model for resistance to *E. turcicum* in the sorghum conversion panel

Dataset	2016 field	2017 field	2016 & 2017 combined	2018 Greenhouse	2018 Greenhouse
	AUDPC	AUDPC	AUDPC	AUDPC	IP
Line	< 0.0001	< 0.0001	< 0.0001	0.2187	0.0464
Set	0.2238	0.1532	0.1031	0.7344	0.252
Rep^a^	0.1187	0.2854	0.0862	–	–
Block^b^	< 0.0001	0.1240	< 0.0001	0.1853	0.1699
En^c^	–	–	0.4955	–	–
Line*Env	–	–	0.0923	–	–

^a^ Rep- replication nested within set

^b^ Block is nested within replication within set

^c^ Env- environment

**Table 2:** Pearson correlation coefficients between the area under the disease progress curve, incubation period and flowering time

Correlation	2017AUDPC	2018IP	Flowering Time
2016AUDPC	0.52^***^	−0.18^***^	0.03
2017AUDPC		−0.09^**^	0.01
2018IP			0.07*

^***^Significant at *P* < 0.01

^**^Significant at *P* < 0.05

^*^Significant at *P* < 0.10

Sorghum consists of five subpopulations, and we examined the average resistance in each group. We did not detect significant differences between subpopulations (Additional file 3: Figure S1). Kafir had the highest average resistance. All groups had highly susceptible lines, indicating that there is no single race that is uniformly resistant. Instead, all races contain alleles that contribute both resistance and susceptibility.

### Significant SNPs associated with disease resistance to *E. turcicum* in sorghum

**Fig. 1:**
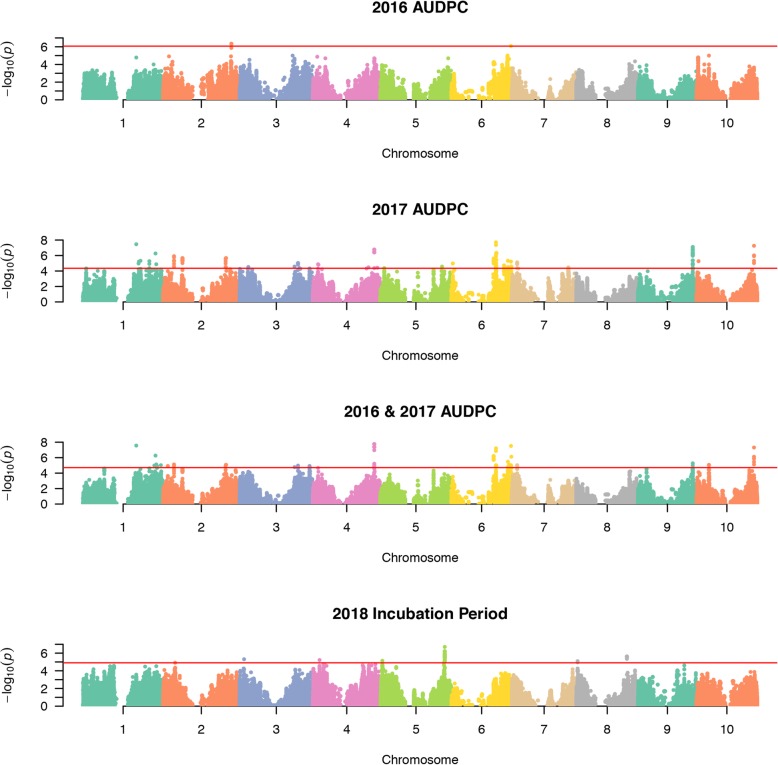
Manhattan plots for genome-wide association mapping. The panels show the results from the 2016 AUDPC, 2017 AUDPC, 2016 and 2017 combined, and the 2018 incubation period datasets

**Fig. 2:**
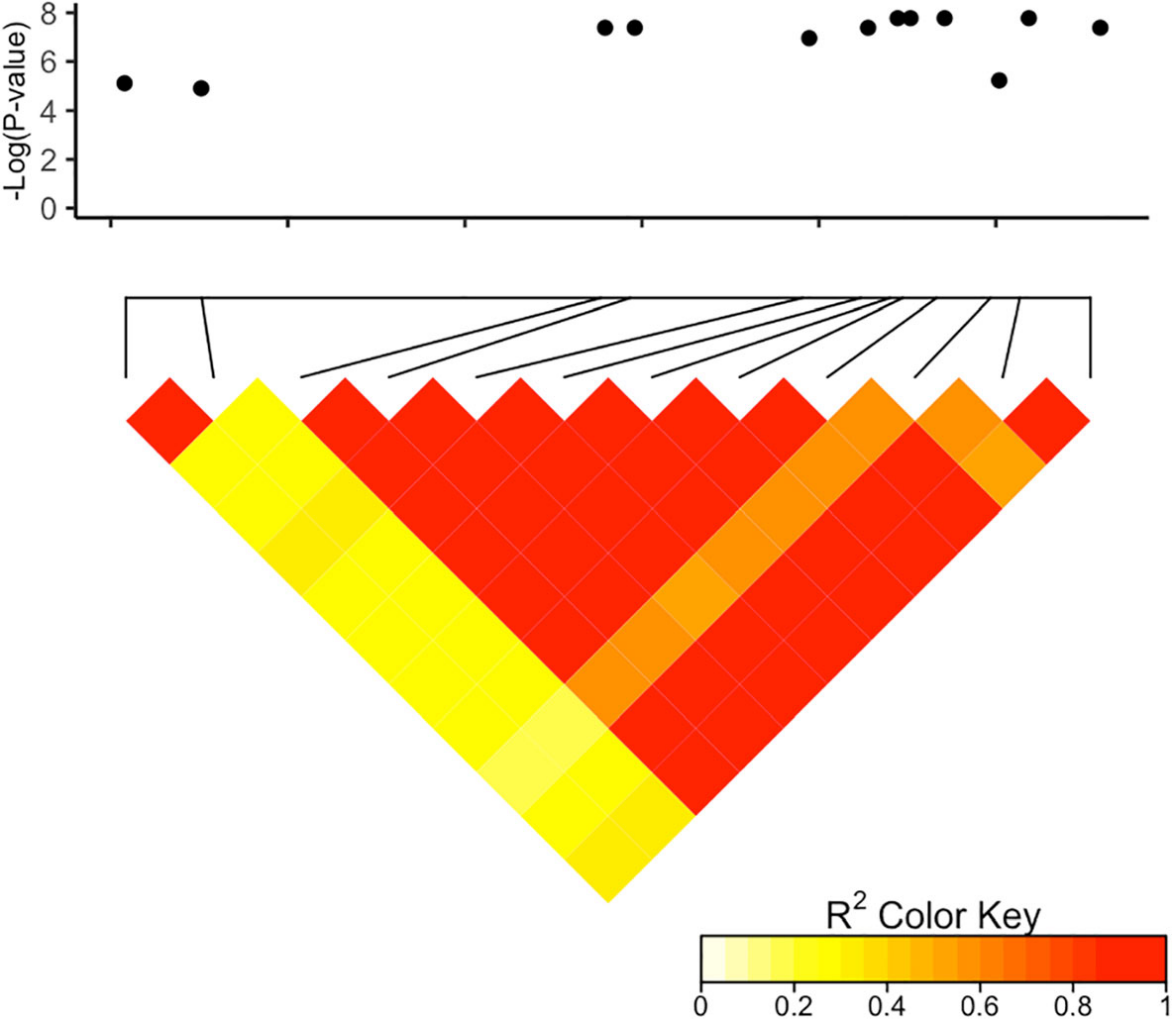
Linkage disequilibrium (LD) plot for the significant SNPs in the chromosome 4 62 Mb region. The Manhattan plot for the region is shown above and the linkage disequilibrium shown below. Only the significant SNPs from the association mapping analysis in the region are shown in the Manhattan plot. In the LD plot, the R^2^ values between significant SNPs are shown. Red indicates high amounts of linkage disequilibrium, while yellows indicates low linkage disequilibrium

We found that many loci are involved in resistance to SLB. We identified 3, 152, 66 and 43 significant markers using the 2016 AUDPC, 2017 AUDPC, 2016 and 2017 combined and the 2018 IP datasets, respectively (Fig. 1). We dectected significant SNPs (FDR <  0.10) on all chromosomes. The region on chromosome 4 spanning from 62,185,882 to 62,289,470 bp harbored the most significant associations in the combined dataset (Fig. 2). Chromosome 6 harbored the most significant hit in the 2017 dataset. Most significant SNPs from the IP analysis were located on chromosome 5, approximately 2 Mb from significant associations from the 2017 dataset.

### Genes involved in sorghum resistance to *E. turcicum*

Using the BTx623 reference sequence, candidate genes were identified based on the physical locations of significant SNPs. In total, we identified 113 unique genes (Additional file 1: Table S1). The top 10 genes, based on FDR-adjusted *P*-values, are shown in Table 3. A total of 23 significant SNPs were identified on chromosome 4 at approximately 62 Mb. The implicated region is 103 Kb and contains 11 genes. It was implicated by both the 2017 and the combined datasets. We examined LD within the region and found there are two LD blocks that had significant associations within the region (Fig. 2). There are several genes possibly involved in plant defense located within the 103 Kb region, including the sorghum ortholog of oxidative stress 3 (﻿Sobic.004G279700.1), ﻿tobamovirus multiplication protein 3, ﻿a heavy metal-associated domain containing protein, and a protein phosphatase. There are significant SNPs in the oxidative stress 3 ortholog and the protein phosphatase.

**Table 3:** The top 10 most significant genes from the genome-wide association mapping

Chr	Position	P-value	Dataset	Type	Gene ID	Arabidopsis annotation
4	62,234,452	1.67E-08	16&17	Intergenic	Sobic.004G279601	Histone superfamily protein
4	62,235,175	1.67E-08	16&17	Intergenic	Sobic.004G279700	Oxidative stress 3
4	62,241,862	1.67E-08	16&17	Genic	Sobic.004G279800	N/A
1	53,698,562	2.92E-08	16&17	Intergenic	Sobic.001G276000	Peptide transporter 2
6	60,634,058	3.17E-08	16&17	Intergenic	Sobic.006G275100	Manganese tracking factor for mitochondrial SOD2
4	62,217,921	4.13E-08	16&17	Intergenic	Sobic.004G279400	Regulator of chromosome condensation (RCC1) family with FYVE zinc finger domain
4	62,219,598	4.13E-08	16&17	Intergenic	Sobic.004G279500	N/A
10	57,920,894	4.87E-08	16&17	Intergenic	Sobic.010G236400	Ubiquitin C-terminal hydrolases superfamily protein
6	45,334,706	6.01E-08	16&17	Genic	Sobic.006G084300	P-loop containing nucleoside triphosphate hydrolases superfamily protein
6	45,346,069	1.36E-07	16&17	Intergenic	Sobic.006G084400	Thylakoidal ascorbate peroxidase

Several of the candidate genes in other regions are implicated in plant defense, including a wound-responsive family protein, as well as a glutathione S-transferase-encoding gene. Auxin response was implicated with an auxin efflux carrier protein and an auxin response factor included in the list. Signaling is also implicated with a mitogen-activated protein kinase, among several other protein kinases. Among the protein kinases implicated, one had a leucine-rich domain and a second had a wall-associated kinase-encoding domain. Also, a gene encoding an NB-ARC domain containing disease resistance protein was included. The oxidative stress-related process is implicated with a peroxidase superfamily protein-encoding gene. A phytoene desaturase-encoding gene, which is key in carotenoid, chlorophyll, and gibberellic acid (GA) biosynthesis, is implicated.

**Fig. 3:**
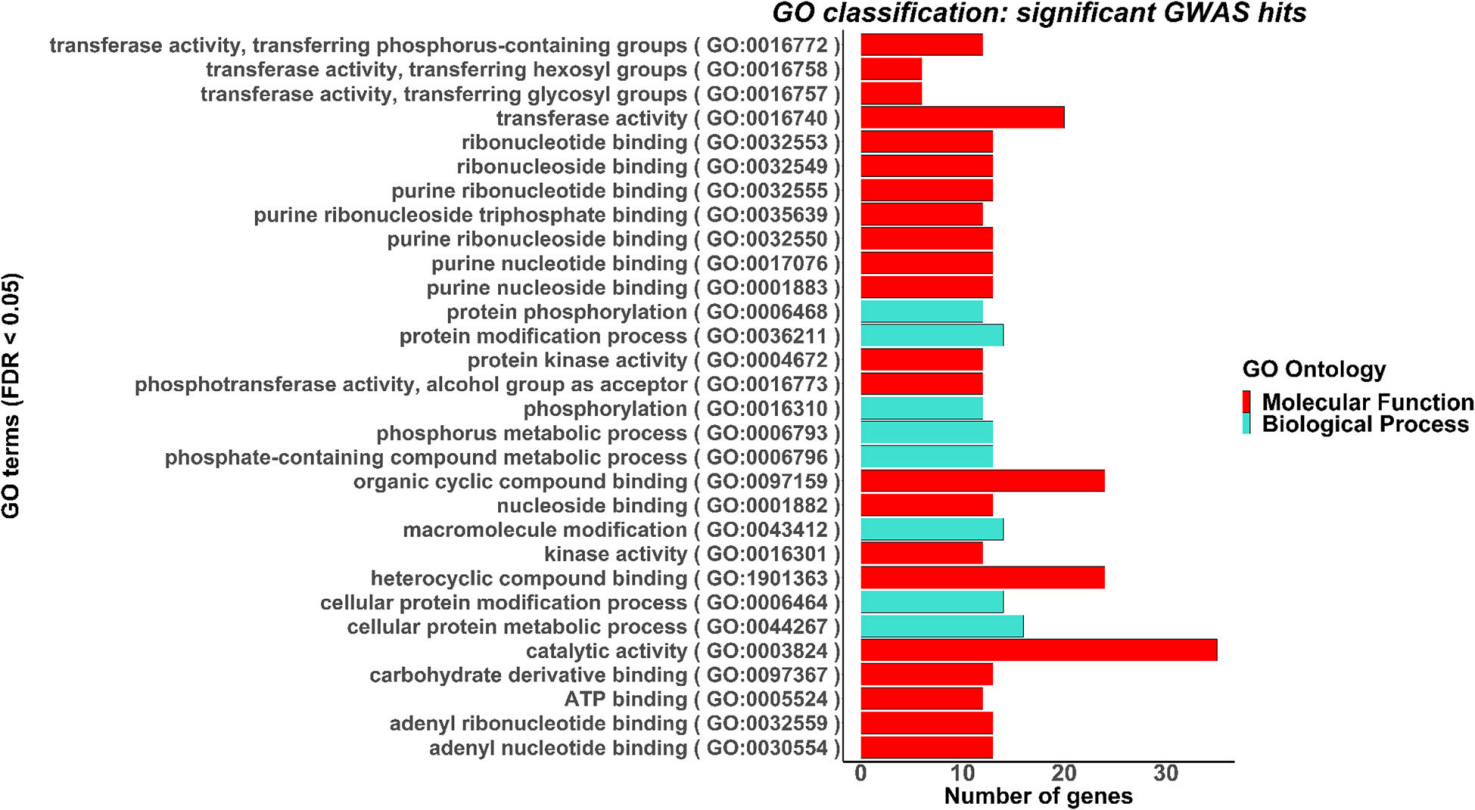
Singular enrichment analysis (SEA). The SEA was conducted using agriGO v2.0. The number of genes in significantly enriched categories in biological processes (blue) and molecular functions (red) are shown

The SEA to assess the functional significance of the candidate genes identified 30 significantly enriched GO terms in the GWAS, which included 8 in biological processes and 22 in molecular function. GO terms enriched in biological processes mostly included protein phosphorylation, protein modification process, protein metabolic process, and phosphorous metabolic process (Fig. 3). GO terms enriched in molecular function included kinase activity, phosphotransferase activity, ATP binding, heterocyclic compound binding, and catalytic activity (Fig. 3).

### Comparison between maize and sorghum resistance to *E. turcicum*

The genetic architecture of resistance in sorghum is similar to that of maize, with many loci involved. We curated a list of 36 maize genes with the strongest support for a role in *E. turcicum* resistance based on previous mapping studies (Additional file 2: Table S2) [12–14, 16, 23–26]. We identified the sorghum orthologs of these maize genes and compared them with our association mapping results. We observed long-distance linkage disequilibrium in the SCP of up to 1 Mb and so considered any associations within 1 Mb of a maize-derived candidate gene (Additional file 4: Figure S2). Of the 36 candidate sorghum genes based on synteny with maize candidates, 12 were within 1 Mb of a significant association (Additional file 2: Table S2). To determine whether that was significantly more genes than expected by chance, we conducted a permutation test. We selected 36 random genes and found how many of those genes were near significant associations. Based on the permutation test, we concluded that our finding of 12 genes within 1 Mb of a significant association is highly significant (*P* <  0.01).

In the comparative analysis, the closest candidate ortholog gene was a zinc finger that was 195 kb from the closest sorghum association [23]. A remorin (SORBI_3001G460300) that was implicated in maize for resistance to NCLB [12] was 394 Kb from a significant association in sorghum. Additionally, the same gene classes that have been implicated in maize were implicated in sorghum as well. A GST-encoding (Sobic.006G085100) gene was implicated in sorghum, and a GST has been implicated in maize for its involvement in multiple disease resistance [14].

## Discussion

We developed a robust high-throughput method to screen sorghum in the field for SLB. Using this method we identified significant differences among genotypes and several significant associations, hence demonstrating its utility. We found that kafir was, on average, the most resistant, which is consistent with a previous study where kafir types were the most resistant [5].

Environmental conditions affected disease development, in particular, field versus greenhouse. We found agreement in the significant associations between the field-based datasets. In 2016, the weather was more conducive to disease development, and more disease was observed. This may account for some of the differences we observed between the 2016 and the 2017 results. Incubation period was the more robust phenotypic measure from the greenhouse study. We did not conduct a combined greenhouse field GWAS because of the inherent differences between the phenotypic measures and lack of correlation between the two environments. The lack of correspondence between field and greenhouse has also been observed in other studies involving *E. turcicum*, specifically in maize where there are NCLB QTL that are effective in the field but not in the greenhouse [27].

Several resources have been developed for genome-wide association mapping in sorghum [22, 28, 29]. Association mapping has been used in sorghum for diverse traits and has been successfully used to identify genes that are known to underlie given traits [30, 31]. Here we used the SCP because all lines flower in central Illinois, and plant height is relatively uniform. These are important factors in evaluating the panel for a disease that is foliar and intensifies after flowering. It is important to note that the design of the SCP prevents the detection of some genomic regions because of the crossing scheme that was used to generate the SC lines [22]. Thus, we would not have detected associations near those loci that are fixed in this population.

There is preliminary evidence that suggests there may be a major gene segregating in the SCP. The bimodal distribution of the IP data suggests that there may be genes in this population acting to delay the onset of disease symptoms. The NCLB major gene *HtN1* prolongs incubation time and latent period [32]. *HtN1* has been cloned in maize and encodes a wall-associated kinase [24]. The closest significant association near the sorghum ortholog of the wall-associated kinase was 459 Kb from the wall-associated kinase [24]. Martin et al. found that maize major genes are present in sorghum [20], and race structure has been observed in sorghum *E. turcicum* isolates [5]. It is important to note that not all the major genes have been cloned in maize and the uncloned genes may be conferring resistance in this panel. Further investigation in biparental populations where the parents differ for incubation period is warranted to determine whether a major gene delaying disease onset is present in this population and whether *HtN1* is present in sorghum.

We identified 113 candidate genes in this study (Additional file 1: Table S1) using a stringent threshold and had highly significant FDR-adjusted *P*-values, indicating they are likely to be true positive results. Furthermore, regions were indicated with several significant associations clustering within an interval, which could be indicative of long-range LD or multiple genes underlying the QTL. It is common for multiple genes physically linked to underlie resistance to this disease [12, 13]. Interesting candidate genes in the chromosome 4 region include a protein phosphatase and the sorghum ortholog of oxidative stress 3. Further work is needed to follow up on these genes and discern their role in SLB resistance.

Several interesting candidate genes were identified through the GWAS, and several biological processes including protein phosphorylation known to be involved in plant defense were implicated through the SEA. A phytoene desaturase (PDS)-encoding gene (Sobic.006G177400) was a candidate based on the mapping. Disruption of a PDS enhanced viral accumulation [33], and further investigation of this gene is merited. An NB-ARC domain containing disease resistance protein (Sobic.002G053300) was identified as a candidate gene. Resistance genes often contain a NB-ARC signaling domain [34], and the complete resistance observed in some lines in our population could indicate that there are major genes present in the population. One of the most significant candidate genes implicated by the IP association mapping is an F-box encoding gene. F-box genes are known to regulate R gene expression [35]. There were several other interesting associations in the IP dataset, including the sorghum ortholog of chloroquine-resistance transporter-like transporter 2. In *Arabidposis*, mutants lacking this gene were hypersensitive to *Phytophthora* infection [36].

We found that genetic architecture of resistance in sorghum to be similar to that of maize, with many loci involved. Complete resistance in maize to *E. turcicum* is rare [14, 37], but in this study, we found several lines that were completely resistant. This may be due to pathogen strains used in this study or the environment being less conducive to infection. In any case, sorghum may be more resistant to *E. turcicum* than maize and harbors alleles for resistance.

We found evidence of shared genetic regions for resistance between maize and sorghum for diseases caused by *E. turcicum*. This is contrast to studies in other systems. For example, resistance QTL in ryegrass and cereals for fungal pathogens did not coincide on a genome-wide level [38]. However, it is important to note that in this study we were examining resistance to the same fungal species across plant hosts. At the gene level, there are examples of quantitative disease genes, such as *POQR* that underwent convergent evolution and confer resistance in multiple hosts [39] and *Lr34* that conferred resistance to several diseases in wheat, maize, and sorghum [40–43]. Further work is required to determine whether the same genes underlie resistance to *E. turcicum* in maize and sorghum.

## Conclusions

In summary, this is the first study using genome-wide association mapping to identify genomic regions associated with SLB resistance. The SCP includes lines highly resistant to SLB. This will help improve breeding for resistance in sorghum, as markers were identified that could be used to breed resistant varieties. We identified 113 candidate genes, including genes with known roles in plant defense and several genes that are implicated in major gene resistance. We compared resistance in maize and sorghum and found a similar genetic architecture of resistance in both crops. We find evidence of shared resistance mechanisms between maize and sorghum with 12 candidate sorghum genes falling within 1 Mb of sorghum orthologs of known maize resistance gene.

## Methods

### Phenotyping

#### Plant materials

We evaluated the SCP [22] for SLB at the Crop Sciences Research and Education Centers in Urbana, IL in the field in 2016 and 2017 and at the Plant Care Facility in Urbana, IL in 2018 in the greenhouse. The conversion lines were initially generated by backcrossing an elite donor to exotic progenitor lines four times with selection at the F_2_ generation for dwarfed and photoperiod-insensitive plants [21]. Seed for SC lines was initially obtained from the USDA-ARS Cropping Systems Research Laboratory (Lubbock, TX, USA). Each line of the SCP was assigned to five sets based on flowering times, as a strong relationship has been reported between flowering time and resistance to *E. turcicum* [14]. Sets were independent of subpopulation.

#### Experimental design

For the field experiments, an incomplete block design with two replicates was created for each set using the R package “*agricolae”* [44, 45], and each block was augmented with one susceptible check line (Tx623) in a random position. Each line was planted in a single-row plot and standard agronomic practices for central Illinois were followed. Before planting seed was treated with Apron (mefenoxam; Syngenta, Switzerland) and Concep (Fluxofenim; Syngenta, Switzerland). Plots were machine-planted at a density of 50 seeds/row and were with 3.65 m long with 0.91-m alleys. We planted a total of 705 and 679 lines in 2016 and 2017, respectively. Fewer lines were evaluated in 2017 due to seed availability.

For the greenhouse experiment, one replication was evaluated in an augmented design with two check lines, Tx623 (susceptible) and SC0283 (resistant), included in each block. The greenhouse evaluations were carried out in Urbana, IL in 2018, using 596 lines with one plant per line in a one-gallon pot filled with general purpose potting mix. The conditions were set to a 12/12-h light-dark cycle and 30/20 °C day-night temperature.

#### Disease screening

We used the three *E. turcicum* isolates 15st003, 15st008, and 16st001, obtained from sorghum leaves in Illinois, to inoculate field-grown plants using solid inoculum. To generate the solid inoculum, isolates were transferred from glycerol stocks stored at − 80 °C to lactose-casein hydrolysate agar (LCA) and incubated at room temperature with a 12/12-h light-dark cycle for 2–3 weeks. The solid sorghum substrate was prepared by mixing 2200 ml untreated sorghum grain with 1375 ml distilled water in autoclave bags. The grain was soaked overnight and then autoclaved twice, for 20 min each time. The autoclaved grain was inoculated with an *E. turcicum* spore suspension prepared by flooding each LCA plate with about 8 ml of ddH_2_O, dislodging spores with glass rods and pipetting approximately 5 ml of undiluted spore suspension into each bag. Each bag was cultured with a single isolate. The inoculum was incubated at room temperature for 2–3 weeks with a 12/12-h light-dark cycle. Grain was redistributed daily to avoid the formation of clumps. Bags with different isolates were mixed immediately preceding inoculations to equalize spore concentrations across bags. Plants were inoculated at the 5–6 leaf stage by applying approximately ¼ teaspoon (1.5 mL) of sorghum grains colonized with *E. turcicum* in the whorl.

For the greenhouse experiment, four-week-old plants were inoculated with 0.5 ml of *E. turcicum* liquid inoculum placed in the whorl [27]. We cultured the same fungal strains on LCA plates, as described above, but adjusted the suspension to a concentration of 4 × 10^3^ conidia per ml. Following inoculation, we placed plants in a mist chamber and high humidity was maintained overnight with overhead misting for 10 s every 15 min.

#### Disease assessment

Diseased leaf area (DLA) was assessed visually and ratings ranged from 0 to 100 with 5% increments [46]. A score of 0 indicated that all plants in the plot were healthy and no lesions were observed, while 100 denoted that all plants in the plot were completely necrotic. Diseased leaf area (DLA) was evaluated on a per-plot basis three times after flowering with an interval of 7 days. For the greenhouse, we evaluated the primary DLA [47] on the inoculated leaf at 14, 21 and 28 days post-inoculation. Additionally, plants were checked for lesion formation on a daily basis and incubation period (IP) was recorded as the number of days post inoculation when the first lesion appeared.

### Genotyping

A dataset of 107,421 SNPs (hereafter referred to as target set) scored using genotyping-by-sequencing was obtained from Fernandes et al. [48] and Thurber et al. [22]. In order to increase the marker density for the target panel, a whole-genome re-sequencing dataset (hereafter referred to as the reference genotype set) was used for imputing un-typed SNPs [49]. The reference set was composed of 239 individuals and 5,512,653 SNPs anchored to the *Sorghum bicolor* reference genome version 3.1 (https://phytozome.jgi.doe.gov) [50]. We filtered the reference set for heterozygosity (> 10%), SNP coverage (<4X) and missing genotypes (> 40%). Additionally, SNPs with minor allele count < 3 and depth < 3 were also filtered out before the imputation. The final reference set included 239 individuals and 4,268,905 SNPs.

Before imputation, the target and reference sets were compared using conform-gt [51]. This step excluded target SNPs not present in the reference genotypes and adjusted the genomic position and chromosome strand to match the target and reference sets. Thus, the set of 34,498 target set SNPs included for imputation had a minor allele frequency > 1% and positions matching the reference panel. Un-typed SNPs were imputed by chromosome, using option gt, window = 80,000 bp, overlap = 10,000 bp and ne = 150,000. After filtering, Beagle version 4.1 was used to impute missing genotypes (option “gtgl”), followed by a phasing (option “gt”) step [52]. We used a window of 1500 bp and an overlap of 500 bp for both steps. The genotypic dataset was pruned using plink based on linkage disequilibrium by removing variants with r^2^ values greater than 0.9, using a window size of 20 and step size of 5 SNPs [53]. The markers were then filtered for a minor allele frequency of 0.05 using GAPIT [54]. We conducted the association analysis for the field datasets using GAPIT version 3.0 [54]. A total of 338,651 markers were included in the analysis.

### Data analysis

Area under disease progress curve (AUDPC) was calculated from the DLA data using the absolute method with the “audpc” function from R package ‘*agricolae*’ [44, 45]. Linear models were run using the PROC MIXED function implemented in SAS version 9.4 (SAS Institute Inc., Cary, NC), and all factors were fit as random effects. Each year was analyzed individually, as well as the combined field data. Field and greenhouse data were not combined due to the inherent differences between the field and greenhouse environments. Initially, models were fit that included design factors and line (Table 1). For the field datasets, set was nested within year, replication was nested within set within year, and block was nested within replication within set. Significance of random factors to include in the models was determined using Wald’s Z-test statistics implemented using the restricted maximum likelihood (REML) method [55]. Additionally, likelihood ratio tests were conducted to determine whether to include factors in the models. The 2016 AUDPC model included line, set, block and replication. The 2017 AUDPC model included line and set. The combined 2016 and 2017 model included line, year, set, rep, block, environment, and the year by environment interaction. Best linear unbiased predictors (BLUPs) were calculated for the 2016, 2017, 2018 IP and the combined 2016 and 2017 datasets. Further analysis was not conducted for 2018 AUDPC, as line was not significant in the analysis. The 2018 IP dataset was divided into two classes based on the BLUPs. Lines with effects less than 0 were considered as resistant, and lines with effects greater than 0 were considered susceptible. The phenotypic data is available in Additional file 5: File S1.

The “CMLM” method was used to conduct the GWAS using GAPIT [54], and a total of four principal components were included. A false discovery rate of 10% was used to determine whether associations were significant [56]. Because the IP dataset was categorical, we employed logistic regression to conduct association mapping using plink version 1.9 [53]. The principal components, as calculated by GAPIT, were included in the plink analysis.

Pearson correlations for flowering time, plant height and subpopulation were conducted using the “rcorr” function in the “Hmisc” package [57] in R. Data for flowering time and plant height were obtained from Thurber et al. [22]. A Tukey’s HSD test was conducted using the ‘*agricolae*’ package [44] in R to determine whether there were significant differences in the combined field data between different subpopulations.

### Candidate gene selection

The physical proximity of significant associations to genomic features was used to identify candidate genes. The BEDTools toolkit was used to identify candidate genes based on significant SNP positions [58, 59]. If significant SNPs were genic, the gene containing the SNP is reported as the candidate gene. If the SNP was intergenic, the closest gene feature is reported as the candidate gene. The functional significance of the candidate genes were determined through singular enrichment analysis (SEA) using agriGO v2.0 [60].

### Maize candidate genes and syntenic sorghum genes

We curated a list of candidate maize resistance genes based on previous studies [12, 13, 16, 23, 24]. The sorghum syntenic orthologs of the curated maize candidate genes were obtained using the methodology described in [61]. To determine whether the number of orthologs close to associations in sorghum was significant, a permutation test was conducted. We randomly selected 36 genes and determined how many of those genes were within 1 Mb of significant associations. We conducted 1000 iterations of this test.

## Supplementary information

**Additional file 1: Table S1.** All genes implicated by genome-wide association mapping. (CSV 50 kb)

**Additional file 2: Table S2.** The list of candidate maize genes and their sorghum orthologs. (CSV 6 kb)

**Additional file 3: Figure S1.** The boxplot of combined 2016 and 2017 AUDPC for five subpopulations. The intercept is not added to the BLUPs. No significant differences were detected between the subpopulations.

**Additional file 4: Figure S2.** Long-range linkage disequilibrium in the sorghum conversion panel.

**Additional file 5: File S1.** Phenotypic data for the sorghum conversion panel. The data are presented as BLUPs for each environment and the combined environments. (CSV 28 kb)

## Data Availability

Datasets supporting the conclusions of this article are included within the article (and its additional files). The genotypic dataset is available through Figshare (doi: 10.6084/m9.figshare.11288204).

## Abbreviations

GWAS: Genome-wide association study
LD: Linkage disequilibrium
NCLB: Northern corn leaf blight
QTL: Quantitative trait locus
QTN: Quantitative trait nucleotide
SCP: Sorghum conversion panel
SLB: Sorghum leaf blight
SNP: single nucleotide polymorphism

## References

[1] Stukenbrock EH, McDonald BA (2008). The origins of plant pathogens in agro-ecosystems. Annu Rev Phytopathol.

[2] Mueller DS, Wise KA, Sisson AJ, Allen TW, Bergstrom GC, Bosley DB, Bradley CA, Broders KD, Byamukama E, Chilvers MI (2016). Corn yield loss estimates due to diseases in the United States and Ontario, Canada from 2012 to 2015. Plant Health Progress.

[3] Frederiksen RA, Odvody GN (2000). Compendium of sorghum diseases.

[4] Hennessy GG, Demilliano WAJ, Mclaren CG (1990). Influence of primary weather variables on sorghum leaf-blight severity in southern africa. Phytopathology.

[5] Ramathani I, Biruma M, Martin T, Dixelius C, Okori P (2011). Disease severity, incidence and races of *Setosphaeria turcica* on sorghum in Uganda. Eur J Plant Pathol.

[6] Borchardt DS, Welz HG, Geiger HH (1998). Genetic structure of *Setosphaeria turcica* populations in tropical and temperate climates. Phytopathology.

[7] Hamid AH, Aragaki M (1975). Inheritance of pathogenicity in *Setosphaeria turcica*. Phytopathology.

[8] Bunkoed W, Kasam S, Chaijuckam P, Yhamsoongnern J, Prathuangwong S. Sexual reproduction of *Setosphaeria turcica* in natural corn fields in Thailand. Kasetsart J. 2014;48(2):175-182.

[9] Nieuwoudt A, Human MP, Craven M, Crampton BG (2018). Genetic differentiation in populations of *Exserohilum turcicum* from maize and sorghum in South Africa. Plant Pathol.

[10] Nelson R, Wiesner-Hanks T, Wisser R, Balint-Kurti P (2018). Navigating complexity to breed disease-resistant crops. Nat Rev Genet.

[11] Yang Q, He Y, Kabahuma M, Chaya T, Kelly A, Borrego E, Bian Y, El Kasmi F, Yang L, Teixeira P (2017). A gene encoding maize caffeoyl-CoA O-methyltransferase confers quantitative resistance to multiple pathogens. Nat Genet.

[12] Jamann TM, Luo X, Morales L, Kolkman JM, Chung CL, Nelson RJ (2016). A remorin gene is implicated in quantitative disease resistance in maize. Theor Appl Genet.

[13] Jamann TM, Poland JA, Kolkman JM, Smith LG, Nelson RJ (2014). Unraveling genomic complexity at a quantitative disease resistance locus in maize. Genetics.

[14] Wisser RJ, Kolkman JM, Patzoldt ME, Holland JB, Yu J, Krakowsky M, Nelson RJ, Balint-Kurti PJ (2011). Multivariate analysis of maize disease resistances suggests a pleiotropic genetic basis and implicates a GST gene. Proc Natl Acad Sci U S A.

[15] Wisser RJ, Balint-Kurti PJ, Nelson RJ (2006). The genetic architecture of disease resistance in maize: a synthesis of published studies. Phytopathology.

[16] Poland JA, Bradbury PJ, Buckler ES, Nelson RJ (2011). Genome-wide nested association mapping of quantitative resistance to northern leaf blight in maize. Proc Natl Acad Sci U S A.

[17] Welz HG, Geiger HH (2000). Genes for resistance to northern corn leaf blight in diverse maize populations. Plant Breed.

[18] Beshir MM, Okori P, Ahmed NE, Rubaihayo P, Ali AM, Karim S (2016). Resistance to anthracnose and turcicum leaf blight in sorghum under dual infection. Plant Breed.

[19] Sharma R, Upadhyaya HD, Manjunatha SV, Rao VP, Thakur RP (2012). Resistance to foliar diseases in a mini-core collection of sorghum germplasm. Plant Dis.

[20] Martin T, Biruma M, Fridborg I, Okori P, Dixelius C (2011). A highly conserved NB-LRR encoding gene cluster effective against *Setosphaeria turcica* in sorghum. BMC Plant Biol.

[21] Stephens JC, Miller FR, Rosenow DT (1967). Conversion of alien sorghums to early combine genotypes. Crop Sci.

[22] Thurber CS, Ma JM, Higgins RH, Brown PJ (2013). Retrospective genomic analysis of sorghum adaptation to temperate-zone grain production. Genome Biol.

[23] Ding J, Ali F, Chen G, Li H, Mahuku G, Yang N, Narro L, Magorokosho C, Makumbi D, Yan J (2015). Genome-wide association mapping reveals novel sources of resistance to northern corn leaf blight in maize. BMC Plant Biol.

[24] Hurni S, Scheuermann D, Krattinger SG, Kessel B, Wicker T, Herren G, Fitze MN, Breen J, Presterl T, Ouzunova M (2015). The maize disease resistance gene *Htn1* against northern corn leaf blight encodes a wall-associated receptor-like kinase. Proc Natl Acad Sci U S A.

[25] Li YX, Chen L, Li C, Bradbury PJ, Shi YS, Song Y, Zhang D, Zhang Z, Buckler ES, Li Y (2018). Increased experimental conditions and marker densities identified more genetic loci associated with southern and northern leaf blight resistance in maize. Sci Rep.

[26] Van Inghelandt D, Melchinger AE, Martinant JP, Stich B (2012). Genome-wide association mapping of flowering time and northern corn leaf blight (*Setosphaeria turcica*) resistance in a vast commercial maize germplasm set. BMC Plant Biol.

[27] Chung CL, Longfellow JM, Walsh EK, Kerdieh Z, Van Esbroeck G, Balint-Kurti P, Nelson RJ (2010). Resistance loci affecting distinct stages of fungal pathogenesis: use of introgression lines for QTL mapping and characterization in the maize--*Setosphaeria turcica* pathosystem. BMC Plant Biol.

[28] Bouchet S, Olatoye MO, Marla SR, Perumal R, Tesso T, Yu J, Tuinstra M, Morris GP (2017). Increased power to dissect adaptive traits in global sorghum diversity using a nested association mapping population. Genetics.

[29] Brenton ZW, Cooper EA, Myers MT, Boyles RE, Shakoor N, Zielinski KJ, Rauh BL, Bridges WC, Morris GP, Kresovich S (2016). A genomic resource for the development, improvement, and exploitation of sorghum for bioenergy. Genetics.

[30] Adeyanju A, Little C, Yu J, Tesso T (2015). Genome-wide association study on resistance to stalk rot diseases in grain sorghum. G3 (Bethesda).

[31] Cuevas Hugo E., Prom Louis K., Cooper Elizabeth A., Knoll Joseph E., Ni Xinzhi (2018). Genome-Wide Association Mapping of Anthracnose (Colletotrichum sublineolum ) Resistance in the U.S. Sorghum Association Panel. The Plant Genome.

[32] Raymundo AD, Hooker AL, Perkins JM (1981). Effect of gene *Htn* on the development of northern corn leaf blight epidemics. Plant Dis.

[33] DeBlasio SL, Rebelo AR, Parks K, Gray SM, Heck MC (2018). Disruption of chloroplast function through downregulation of phytoene desaturase enhances the systemic accumulation of an aphid-borne, phloem-restricted virus. Mol Plant-Microbe Interact.

[34] van der Biezen EA, Jones JD (1998). The NB-ARC domain: a novel signalling motif shared by plant resistance gene products and regulators of cell death in animals. Curr Biol.

[35] Gou M, Shi Z, Zhu Y, Bao Z, Wang G, Hua J (2012). The F-box protein CPR1/CPR30 negatively regulates R protein SNC1 accumulation. Plant J.

[36] Maughan SC, Pasternak M, Cairns N, Kiddle G, Brach T, Jarvis R, Haas F, Nieuwland J, Lim B, Muller C (2010). Plant homologs of the *Plasmodium falciparum* chloroquine-resistance transporter, PfCRT, are required for glutathione homeostasis and stress responses. Proc Natl Acad Sci U S A.

[37] Chung CL, Poland J, Kump K, Benson J, Longfellow J, Walsh E, Balint-Kurti P, Nelson R (2011). Targeted discovery of quantitative trait loci for resistance to northern leaf blight and other diseases of maize. Theor Appl Genet.

[38] Jo YK, Barker R, Pfender W, Warnke S, Sim SC, Jung GH (2008). Comparative analysis of multiple disease resistance in ryegrass and cereal crops. Theor Appl Genet.

[39] Badet T, Voisin D, Mbengue M, Barascud M, Sucher J, Sadon P, Balague C, Roby D, Raffaele S (2017). Parallel evolution of the POQR prolyl oligo peptidase gene conferring plant quantitative disease resistance. PLoS Genet.

[40] Schnippenkoetter W, Lo C, Liu GQ, Dibley K, Chan WL, White J, Milne R, Zwart A, Kwong E, Keller B (2017). The wheat Lr34 multipathogen resistance gene confers resistance to anthracnose and rust in sorghum. Plant Biotechnol J.

[41] Sucher J, Boni R, Yang P, Rogowsky P, Buchner H, Kastner C, Kumlehn J, Krattinger SG, Keller B (2017). The durable wheat disease resistance gene *Lr34* confers common rust and northern corn leaf blight resistance in maize. Plant Biotechnol J.

[42] Spielmeyer W, Singh RP, McFadden H, Wellings CR, Huerta-Espino J, Kong X, Appels R, Lagudah ES (2008). Fine scale genetic and physical mapping using interstitial deletion mutants of Lr34 /Yr18: a disease resistance locus effective against multiple pathogens in wheat. Theor Appl Genet.

[43] Krattinger SG, Lagudah ES, Spielmeyer W, Singh RP, Huerta-Espino J, McFadden H, Bossolini E, Selter LL, Keller B (2009). A putative ABC transporter confers durable resistance to multiple fungal pathogens in wheat. Science.

[44] De Mendiburu F (2014). Agricolae: statistical procedures for agricultural research.

[45] R Core Team (2018). R: a language and environment for statistical computing. R Foundation for statistical Computing.

[46] Poland JA, Nelson RJ (2011). In the eye of the beholder: the effect of rater variability and different rating scales on QTL mapping. Phytopathology.

[47] Chung CL, Jamann T, Longfellow J, Nelson R (2010). Characterization and fine-mapping of a resistance locus for northern leaf blight in maize bin 8.06. Theor Appl Genet.

[48] Fernandes SB, Dias KOG, Ferreira DF, Brown PJ (2018). Efficiency of multi-trait, indirect, and trait-assisted genomic selection for improvement of biomass sorghum. Theor Appl Genet.

[49] Valluru R, Gazave EE, Fernandes SB, Ferguson JN, Lozano R, Hirannaiah P, Zuo T, Brown PJ, Leakey ADB, Gore MA (2019). Deleterious mutation burden and its association with complex traits in sorghum (*Sorghum bicolor*). Genetics.

[50] McCormick RF, Truong SK, Sreedasyam A, Jenkins J, Shu S, Sims D, Kennedy M, Amirebrahimi M, Weers BD, McKinley B (2018). The *Sorghum bicolor* reference genome: improved assembly, gene annotations, a transcriptome atlas, and signatures of genome organization. Plant J.

[51] Browning SR, Browning BL (2007). Rapid and accurate haplotype phasing and missing-data inference for whole-genome association studies by use of localized haplotype clustering. Am J Hum Genet.

[52] Browning BL, Browning SR (2016). Genotype imputation with millions of reference samples. Am J Hum Genet.

[53] Purcell S, Neale B, Todd-Brown K, Thomas L, Ferreira MA, Bender D, Maller J, Sklar P, de Bakker PI, Daly MJ (2007). PLINK: a tool set for whole-genome association and population-based linkage analyses. Am J Hum Genet.

[54] Tang You, Liu Xiaolei, Wang Jiabo, Li Meng, Wang Qishan, Tian Feng, Su Zhongbin, Pan Yuchun, Liu Di, Lipka Alexander E., Buckler Edward S., Zhang Zhiwu (2016). GAPIT Version 2: An Enhanced Integrated Tool for Genomic Association and Prediction. The Plant Genome.

[55] Littell RC (1996). SAS system for mixed models.

[56] Benjamini Y, Hochberg Y (1995). Controlling the false discovery rate - a practical and powerful approach to multiple testing. J Royal Stat Soc Series B-Stat Methodol.

[57] Harrell F (2019). Package ‘Hmisc’. R package version.

[58] Quinlan AR (2014). BEDTools: The Swiss-Army Tool for Genome Feature Analysis. Curr Protoc Bioinformatics.

[59] Paterson AH, Bowers JE, Bruggmann R, Dubchak I, Grimwood J, Gundlach H, Haberer G, Hellsten U, Mitros T, Poliakov A (2009). The *Sorghum bicolor* genome and the diversification of grasses. Nature.

[60] Tian T, Liu Y, Yan HY, You Q, Yi X, Du Z, Xu WY, Su Z (2017). agriGO v2.0: a GO analysis toolkit for the agricultural community, 2017 update. Nucleic Acids Res.

[61] Zhang Y, Ngu DW, Carvalho D, Liang ZK, Qiu YM, Roston RL, Schnable JC (2017). Differentially regulated Orthologs in Sorghum and the subgenomes of maize. Plant Cell.

