# Supplementary figures and images for: Conserved defense responses between maize and sorghum to Exserohilum turcicum
Source: BMC Plant Biol. 2020 Feb 10;20:67. doi: 10.1186/s12870-020-2275-z (PMC7011368; doi:10.1186/s12870-020-2275-z)

BLUP

150  
100  
50  
0

Bicolor

Caudatum

Durra

Guinea

Kafir

Race

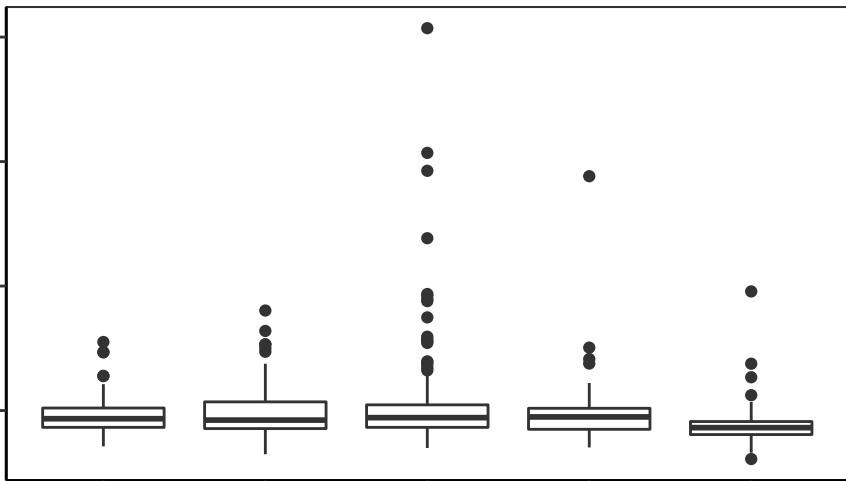

Supplement: Supplementary file 3 — Additional file 3: Figure S1. The boxplot of combined 2016 and 2017 AUDPC for five subpopulations. The intercept is not added to the BLUPs. No significant differences were detected between the subpopulations. [file 12870_2020_2275_MOESM3_ESM.pdf]

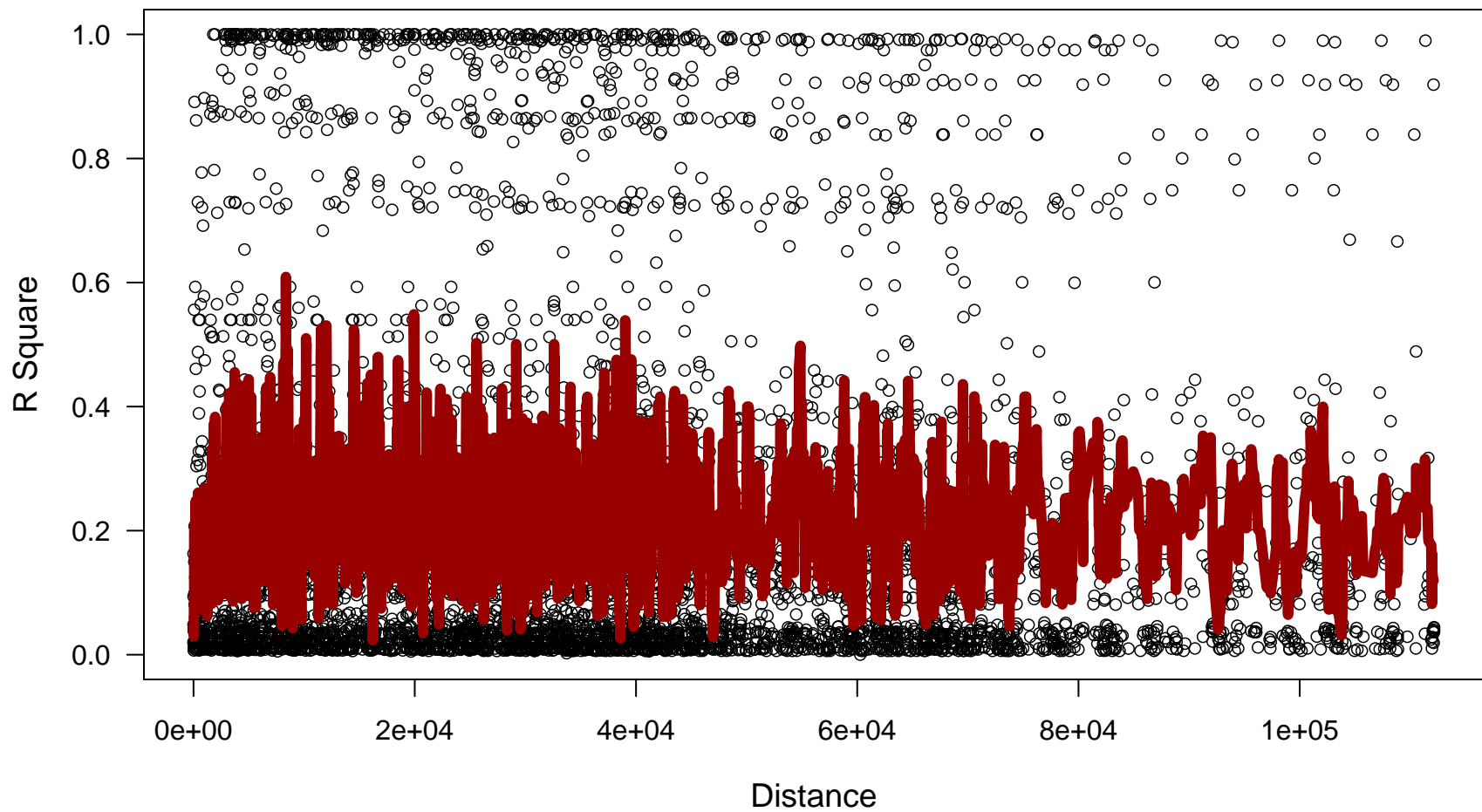

Supplement: Supplementary file 4 — Additional file 4: Figure S2. Long-range linkage disequilibrium in the sorghum conversion panel. [file 12870_2020_2275_MOESM4_ESM.pdf]
